# Endothelial NOX4 Oxidase Negatively Regulates Inflammation and Improves Morbidity During Influenza A Virus Lung Infection in Mice

**DOI:** 10.3389/fcimb.2022.883448

**Published:** 2022-05-04

**Authors:** Keshia S. Hendricks, Eunice E. To, Raymond Luong, Felicia Liong, Jonathan R. Erlich, Ajay M. Shah, Stella Liong, John J. O’Leary, Doug A. Brooks, Ross Vlahos, Stavros Selemidis

**Affiliations:** ^1^ Department of Pharmacology, Infection and Immunity Program, Biomedicine Discovery Institute, Monash University, Clayton, VIC, Australia; ^2^ School of Health and Biomedical Sciences, Science, Technology, Engineering and Mathematics (STEM) College, Royal Melbourne Institute of Technology (RMIT) University, Bundoora, VIC, Australia; ^3^ King’s College London British Heart Foundation Centre, School of Cardiovascular Medicine and Sciences, London, United Kingdom; ^4^ Discipline of Histopathology, School of Medicine, Trinity Translational Medicine Institute (TTMI), Trinity College Dublin, St. James’s Hospital Dublin, Dublin, Ireland; ^5^ Clinical and Health Sciences, Cancer Research Institute, University of South Australia, Adelaide, SA, Australia

**Keywords:** influenza A virus, endothelial cell, oxidative stress, NADPH oxidase 4(NOX4), inflammation

## Abstract

Endosomal NOX2 oxidase-dependent ROS production promotes influenza pathogenicity, but the role of NOX4 oxidase, which is highly expressed in the lung endothelium, is largely unknown. The aim of this study was to determine if endothelial NOX4 expression can influence viral pathology *in vivo*, using a mouse model of influenza infection. WT and transgenic endothelial NOX4 overexpressing mice (NOX4 TG) were infected intranasally with the Hong Kong H3N2 X-31 influenza A virus (10^4^ PFU; HK x-31) or PBS control. Mice were culled at either 3 or 7 days post-infection to analyse: airway inflammation by bronchoalveolar lavage fluid (BALF) cell counts; NOX4, as well as inflammatory cytokine and chemokine gene expression by QPCR; and ROS production by an L-012-enhanced chemiluminescence assay. Influenza A virus infection of WT mice resulted in a significant reduction in lung NOX4 mRNA at day 3, which persisted until day 7, when compared to uninfected mice. Influenza A virus infection of NOX4 TG mice resulted in significantly less weight loss than that of WT mice at 3-days post infection. Viral titres were decreased in infected NOX4 TG mice compared to the infected WT mice, at both 3- and 7-days post infection and there was significantly less lung alveolitis, peri-bronchial inflammation and neutrophil infiltration. The oxidative burst from BALF inflammatory cells extracted from infected NOX4 TG mice was significantly less than that in the WT mice. Expression of macrophage and neutrophil chemoattractants CXCL10, CCL3, CXCL1 and CXCL2 in the lung tissue were significantly lower in NOX4 TG mice compared to the WT mice at 3-days post infection. We conclude that endothelial NOX4 oxidase is protective against influenza morbidity and is a potential target for limiting influenza A virus-induced lung inflammation.

## Introduction

Severe cases of influenza infection are characterised by an excessive and detrimental inflammatory response, lung damage (Mauad et al., 2010) and cardiovascular complications (Dawood et al., 2012). Airway epithelial cells, macrophages and other inflammatory cells shape the inflammatory response to influenza A virus (IAV) infection, and this can induce significant lung damage. Endothelial cells undergo apoptosis in response to IAV, resulting in increased permeability of the monolayer, which could account for the pulmonary oedema often observed in seriously ill patients (Armstrong et al., 2012). Indeed, the targeting of endothelial cells using the anti-inflammatory agonist, 2-amino-4-(4-heptyloxyphenyl)-2-methylbutanol (AAL-R), can ameliorate the influenza-induced cytokine storm, implying that immune cell infiltration and cytokine production are both orchestrated by lung endothelial cells (Teijaro et al., 2011). If endothelial cells are critical for immune cell activation in the lung, then it was reasoned that the reactive oxygen species (ROS) status in these cells may control the amount of inflammation and pathogenesis during a viral infection.

ROS are also implicated in IAV pathogenesis (Vlahos et al., 2011; Vlahos et al., 2012; Vlahos and Selemidis, 2014). ROS are pleiotropic molecules, which serve both physiological and pathological functions in the context of IAV infections. The cellular source and subcellular compartmentalization of ROS production can impact on their ultimate biological function (Selemidis, 2019). For example, ROS can be generated by inflammatory cells such as neutrophils and macrophages *via* either an endosomal NOX2 NADPH oxidase or by alterations in cell metabolism resulting in mitochondrial ROS generation. Recent work by our group and others have shown that mice deficient in NOX2 have an ameliorated IAV-induced inflammatory response in the airways, which is associated with reduced lung oxidative stress and viral titres (Vlahos et al., 2011). Moreover, we showed that IAV of low to high pathogenicity activate endosomal NOX2, which generated ROS in macrophages (To et al., 2017). The resultant endosomal hydrogen peroxide (H_2_O_2_) modified critical cysteine residues on the TLR7 protein, which can alter signalling and lead to reduced antiviral cytokine production, promoting viral replication and disease pathogenesis (To et al., 2017). In contrast to NOX2, airway epithelial cells also generate significant levels of ROS, including H_2_O_2_ *via* the DUOX 1/2 enzymes, to exert anti-bacterial and antiviral responses, although the mechanisms are not well characterised. IAV infection leads to a DUOX2 up-regulation and DUOX-mediated ROS generation (Strengert et al., 2014). *In vivo* silencing of DUOX increased the viral load after intranasal infection of mice with 2009 pandemic H1N1 influenza virus (Strengert et al., 2014). This suggests that there is a dynamic balance in compartmentalised ROS production, which has the potential to regulate viral pathogenesis.

The endothelium is a major regulator of vascular permeability during viral infection. Therefore the role of enzymatic sources of endothelial ROS underpinning the pathogenesis of IAV infection is worthy of investigation. In this regard, the NOX4 isoform of the NADPH oxidase enzyme family is expressed in at least 100 fold higher amounts than NOX2 in endothelial cells, and is the major source of ROS by these cells (Van Buul et al., 2005). Importantly it has a unique mode of activation and pattern of physiological effects. Unlike NOX2, which requires the assembly of multiple subunits for activation, NOX4 is constitutively active once it forms a heterodimer with p22phox and is thought to be regulated primarily through its expression (Drummond et al., 2011). NOX4 oxidase also differs from the other NADPH oxidases in that, due to its extended E-loop, it directly produces H_2_O_2_ rather than superoxide. These differences in structure and function become important when considering the role of NOX2 and NOX4 in the vasculature. NOX2 and NOX4 have been shown to activate distinct kinase pathways in response to stimulation by agonists in HEK293 cells (Anilkumar et al., 2008). While NOX2 exacerbates oxidative stress, inflammation and infarct volume in mouse models of stroke (Walder et al., 1997; Chen et al., 2011; De Silva et al., 2011), NOX4 was found to be protective (Schroder et al., 2012). NOX4 accounted for approximately 75% of the H_2_O_2_ formed in the vasculature and promoted angiogenesis. NOX4 also limited angiotensin-induced vascular dysfunction, and promoted nitric oxide and heme oxygenase 1 (HO-1) production (Fredenburgh et al., 2007). Furthermore, NOX4 has been shown to have protective anti-inflammatory effects in atherosclerosis and is down regulated in patients with atherosclerosis and diabetes, and in mouse models of atherosclerosis (Gray et al., 2016). Interestingly, overexpressing NOX4 specifically in endothelial cells, resulted in enhanced vasodilation and reduced systolic blood pressure (Ray et al., 2011). The opposing effects of NOX4 and endosomal NOX2 on inflammation and cytokine signalling might therefore be of significance for the exacerbated inflammation and pathogenesis resulting from influenza and other viral infections.

While there have been several studies examining the role of NOX4 in inflammatory pathways in the setting of cardiovascular disease, there has been limited investigation of the role of NOX4 in influenza pathology. To the best of our knowledge, there has been only one study examining the potential role of NOX4 in influenza infection and pathogenesis (Amatore et al., 2015). Increased NOX4 expression was detected in human mucoepidermoid pulmonary carcinoma (NCI-H292) cells that had been infected with the highly pathogenic influenza strain A/Puerto Rico/8/34 H1N1 PR8 (Amatore et al., 2015). Treatment with NADPH oxidase inhibitor diphenyleneiodonium (DPI) and knocking down NOX4 expression with siRNA prevented viral replication in this *in vitro* model of infection. The complexity of cancer cell ROS biology could have a confounding effect on the conclusions from this initial viral study and there have been no *in vivo* studies examining the effect of endothelial NOX4 on influenza pathology in an animal model of pathogenesis. The aim of this study was therefore to determine if endothelial NOX4 expression could influence the amount of airway/lung inflammation, and morbidity in response to IAV infection *in vivo*. We have investigated viral pathogenesis in the endothelial NOX4 overexpressing mouse [2-3 fold increase in endothelial NOX4 expression (Ray et al., 2011)] and WT control mice to examine the differential effect of NOX4 expression on influenza pathogenesis.

## Methods

### Animals

The experiments described in this study were approved by the Animal Experimentation Ethics Committee of Monash University and conducted in compliance with the guidelines of the National Health and Medical Research Council (NHMRC) of Australia on animal experimentation. The mice used were males and aged matched (10-15 weeks) and were given unrestricted access to water and standard mouse chow. The wild type C57BL6J mice were obtained from Monash Animal Services (Monash University, Melbourne), and the endothelial NOX4 overexpressing mice were initially created by Professor Ajay Shah from King’s College London, United Kingdom (Ray et al., 2011).

### Influenza A Virus Stocks

The strain of IAV used was Hong Kong HK x-31 (H3N2), a mildly pathogenic, mouse-adapted strain supplied by Professor John Stambas (School of Medicine, Deakin University, CSIRO) and Professor Patrick Reading (Peter Doherty Institute, The University of Melbourne). The virus was stored at concentration of 7x10^8^ PFU/mL, at -80°C. At the time of infection, the virus was diluted to 2x10^5^ PFU/mL and kept on ice.

### Mouse Model and Bronchoalveolar Lavage

WT and NOX4 TG mice were weighed and then anaesthetised with isoflurane, then infected with HK x-31 (10^4^ PFU) diluted in 50 µL PBS or given 50 µL PBS, intranasally. Mice were monitored and weighed daily, then culled on either day 3 or day 7 post infection with an overdose of ketamine-xylazine (100 mg/kg) *via* intraperitoneal injection. The day 3 time point was chosen as it represents the peak of the viral burden and airways inflammation to IAV with this strain (Vlahos et al., 2011; Selemidis et al., 2013; Yatmaz et al., 2013) whereas at day 7 the virus has almost been completely cleared from the lungs (Vlahos et al., 2011; Selemidis et al., 2013; Yatmaz et al., 2013). The lungs were lavaged with PBS as previously published (To et al., 2017), then removed and weighed. The large left lobe was fixed in 10% formalin for histology and the rest was frozen with liquid nitrogen for RNA extraction.

### Lung Sections

The left lung lobe of each mouse was washed in PBS and fixed in 10% neutral buffered formalin overnight. The lung was then processed in paraffin wax, cut into 3-4 μm thick longitudinal sections and stained with hematoxylin and eosin (H&E) by the Monash Histology Platform. Using light microscopy, the lung sections were scanned and uploaded to the Aperio microscope slide scanner (Leica biosystems, Nussloch, Germany) by the Monash Histology Platform. The images were viewed using Aperio Imagescope software. To assess lung pathology, two blinded independent researchers examined random fields of the H&E stained lung sections and graded the alveolitis, peribronchiolar inflammation and inflammatory cell infiltration on a scale of 0-5 as previously published (Tate et al., 2009). The scale of 0-5 corresponds to no inflammation, very mild, moderate, marked, and severe inflammation, respectively. The peri-bronchial inflammation was graded from five small airways per section and alveolitis in 5 random fields per section.

### BALF Cell Counting and Differentials

Ten µL of the BALF was mixed with 10 µL trypan blue, and 10 µL of the mixture then counted with a Countess Cell Counter (Invitrogen, Carlsbad, United States). 50,000 cells were made up in 200 µL PBS and were attached to slides using a Cytospin centrifuge (Thermo Shandon Cytospin 3). The slides were dried in room temperature, fixed in 100% iso-propanol for 1 minute, and allowed to dry again overnight at room temperature. Slides were then stained with Rapid 1 dye (Amber Scientific, Western Australia, Australia) for four minutes while agitating constantly, and then rinsed thoroughly with water. Slides were then stained with Rapid 2 dye (Amber Scientific, Western Australia, Australia) for four minutes while agitating constantly, and then rinsed with water again. Slides were then submerged in one, 70% ethanol, two, absolute ethanol, three, xylene for 5 minutes, and xylene again for another 5 minutes. Finally, the slides were mounted in DPX mounting medium (Thermofisher, MA, USA) and cover slipped. 500 cells per slide/mouse from 5 random fields were differentiated into macrophages, neutrophils, eosinophils, and lymphocytes by a standard morphological criteria using a light microscope. The differentials were performed in a blinded manner by 2 independent examiners.

### NOX2-Derived ROS Detection by L-012 Chemiluminescence

L-012 chemiluminescence of BALF inflammatory cells was performed as per previously described (Vlahos et al., 2011). 50, 000 cells from each animal were seeded in a 96-well plate in Dulbecco’s Modified Eagle’s Medium (DMEM) (10% FBS) in triplicate. A blank triplicate containing only DMEM was included. When adhered, the cells were washed with Krebs-HEPES (warmed to 37°C). 200 μl of the assay solution (Krebs-HEPES (37°C) containing L-012 (10^−4^ M) and PDB (10^−6^ M)) were added to each well in light-sensitive conditions. Luminescence (relative light units RFU/sec) was measured using a Hidex multi-detection platform. The temperature was set at 37°C, luminescence in each well was measured for one second over 60 cycles.

### Real-Time Quantitative PCR

The right lobe of the lung was crushed under liquid nitrogen and total RNA was extracted from approximately 25 mg of lung tissue using the RNeasy kits (Qiagen, Hilden, Germany) as per manufacturers’ instructions. The extracted RNA was measured using a NanoDrop 1000 spectrophotometer and converted to cDNA using the high capacity cDNA reverse transcriptase kit (Applied Biosystems, California, United States). The mRNA expresssion of CXCL10, CCL3, CXCL1, CXCL2, NOX4 and influenza nucleoprotein was quantified using a TaqMan^®^ gene expression assay (Applied Biosystems). For genes of interest, 100 ng of cDNA was loaded into each well, and 10 ng of cDNA was loaded into each well for the 18S housekeeping gene. The plate was run for 2 minutes at 50°C, 1 hour at 95°C, and alternating between 95°C for 15 seconds and 60°C for a minute for 40 cycles. Fold changes in gene expression was calculated by finding the Δ-threshold cycle by subtracting the CT from the 18S CT for each treatment group, finding the ΔΔ-CT by subtracting the average Δ-CT of respective controls wildtype mice from the Δ-CT for each treatment group, and then calculating 2^-(ΔΔ-CT)^. The viral titre was calculated similarly except the NOX4 TG viral titre was normalised to the NOX4 TG uninfected group.

### Statistical Analysis

Data are expressed as mean ± standard error of the mean (S.E.M), with ‘n’ representing the number of mice per experiment. All statistical analyses were performed using GraphPad Prism (version 8). A one-way ANOVA with Tukey Kramer *post hoc* test was used to determine the statistical differences in lung weights and BALF cell counts between groups. The viral titre and the fold-change in NOX4 expression were analysed with a Students unpaired t-test. The chemiluminescence and body weight data were analysed using a two-way ANOVA with a Tukey Kramer *post hoc* test. The lung histology scoring was analysed with a non-parametric one-way ANOVA with a Kruskal-Wallis. A P value of <0.05 was deemed to be statistically significant.

## Results

### Lung NOX4 Expression and Influenza Viral mRNA Expression

**Figure 1:**
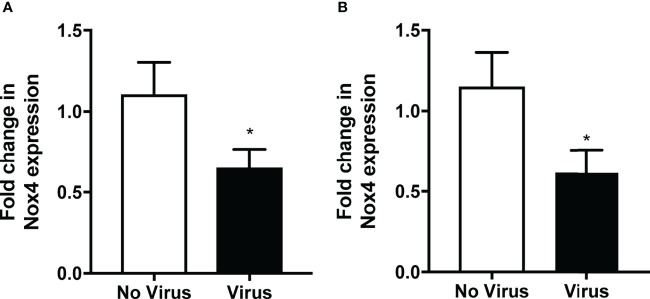
Lung NOX4 expression was decreased in mice infected with IAV. WT mice were infected with HK x31 (10^4^ PFU). Lung NOX4 mRNA expression was measured **(A)**. 3-days and **(B)** 7-days post infection with qPCR. Responses are relative to 18S and expressed as a fold change to the no virus controls. Data shown as mean ± S.E.M and analysed using an unpaired Student’s t-test (n = 6) (*p < 0.05).

**Figure 2:**
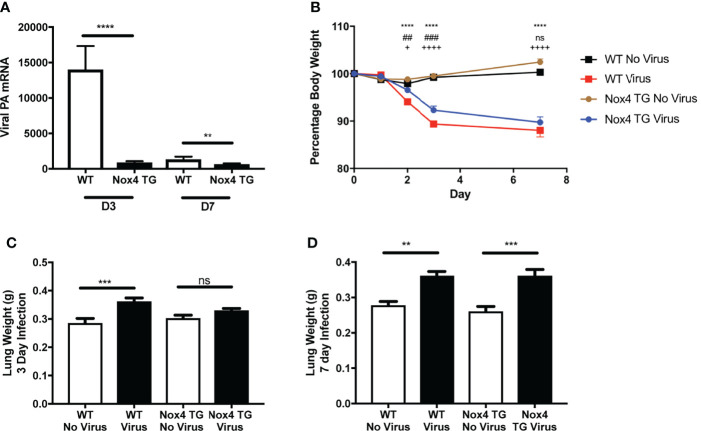
Effect of IAV infection on lung viral mRNA, body weight and lung weights in WT and endothelial NOX4 overexpressing mice. WT and NOX4 TG mice were infected with HK x-31 (10^4^ PFU). Lung viral mRNA for influenza nucleoprotein were measured **(A)** 3- and 7-days post infection. 18S was used as a housekeeping gene. Data shown as mean ± S.E.M and analysed using an unpaired Student’s t-test (n = 6-15). ** and **** represent P < 0.01 and P < 0.0001, respectively. **(B)** Body weight of HK x-31-infected (10^4^ PFU) WT and NOX4 TG mice as a percentage of the pre-infection body weight. Data shown as mean ± S.E.M and analysed using two-way ANOVA with a Tukey Kramer post hoc test. * WT No Virus compared to WT Virus; # WT Virus compared to NOX4 TG Virus; + NOX4 TG No Virus compared to NOX4 TG Virus) (n=8-15). **(C, D)** Lung weight (grams) of WT and NOX4 TG mice at 3-days and 7-days post infection with HK X-31 (104 PFU). Data shown as mean ± s.e.m and analysed using one-way ANOVA with a Tukey Kramer post hoc test (n = 7-15). ** and *** symbols represent P < 0.01; P < 0.001, respectively and ns represents not-significant.

As mentioned previously, NOX4 is constitutively active, and therefore it is suggested that the mRNA expression is reflective of the activity of this enzyme (Drummond et al., 2011). Therefore, we measured the expression of NOX4 at 3- and 7-days post infection to determine whether IAV infections can alter the expression of NOX4 *in vivo*. NOX4 mRNA expression in lung tissue of HK x-31-infected WT mice was significantly reduced at day 3 post infection and at 7-days post infection compared to the uninfected WT mice (**Figures 1A, B**). Interestingly we observed a significant decrease in lung levels of IAV polymerase mRNA in NOX4 TG mouse lungs compared to the WT mice at both 3- and 7-days post infection (**Figure 2A**) suggesting endothelial NOX4 suppresses viral replication.

### Body Weight Loss and Lung Weight Following Influenza A Virus Infection

Bodyweight measurements were recorded daily as an index of disease severity. There was no change in bodyweight in uninfected WT or NOX4 TG mice over the course of 7 days. Starting from day 2 there was a significant weight loss in HK x-31-infected WT mice (**Figure 2B**). NOX4 TG mice had a modest but significant decrease in weight loss compared to the infected WT mice at day 2 and day 3 post infection, but not at day 7 post infection (**Figure 2B**).

Another proxy marker for disease severity is the lung weight, as this is indicative of inflammation and oedema. There was a significant increase in lung weight in Hk x-31-infected WT mice 3- and 7-days post infection compared to uninfected mice (**Figures 2C, D**). There was significantly less lung weight in NOX4 TG mice 3-days post infection but no difference between the HK x-31-infected WT and NOX4 TG mouse lung weights at 7-days post infection (**Figures 2C, D**).

### Lung Inflammation

**Figure 3:**
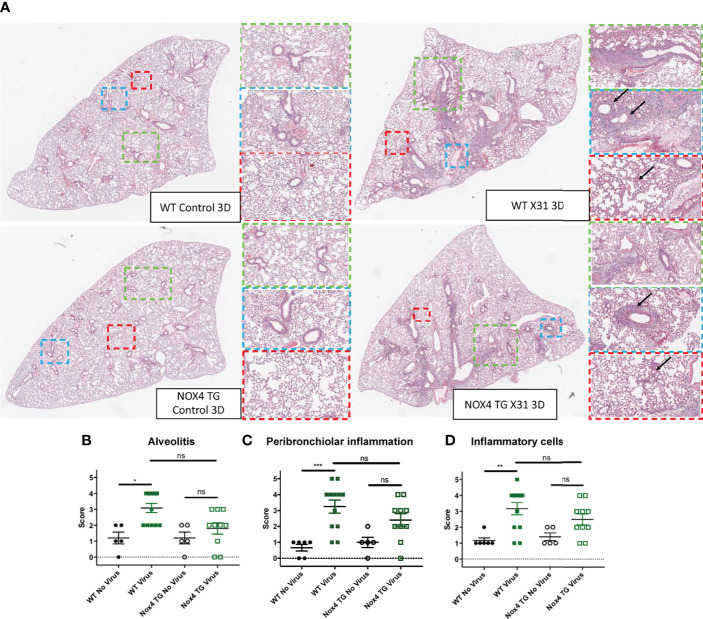
The lungs of IAV-infected NOX4 transgenic mice had less alveolitis, peribronchiolar inflammation and inflammatory cells than IAV infected WT mice 3-days post infection. **(A)** Hematoxylin and eosin (H&E) stained paraffin sections of lungs of WT and NOX4 TG mice 3-days post infection with HK x-31 (10^4^ PFU) or treatment with PBS. **(B)** Alveolitis **(C)** peribronchiolar inflammation and **(D)** inflammatory cell infiltration. Representative images displaying the inflammation in lung that were sectioned longitudinally following H&E staining. The arrows within the blue areas indicate the peri-bronchial inflammation and in the red areas they indicate the alveolitis. Each sample was scored blindly from 0-5 for each individual mouse (higher numbers indicate increased disease severity) from two independent assessors. The representative images were obtained at a x40 magnification using the Aperio Slide Scanning Unit. The images were then viewed with the Aperio image software and different areas of the lung section were subsequently digitally zoomed as shown in the figure. The three different digital magnifications are x1 (green), x3 (blue) and x6(red). Data shown as mean ± S.E.M and analysed using a non-parametric one-way ANOVA with a Kruskal-Wallis post hoc test (n=5-12). *P < 0.05, **P < 0.01, ***P < 0.001, ns represents not-significant.

The degree of cellular infiltrate in the lung and alveolar space is a major contributing factor to influenza virus pathology, and this pathology was scored in H&E stained lung sections to define airway and lung inflammation. There was a significant increase in alveolitis, peribronchiolar inflammation and inflammatory cells in the lungs of WT mice that were infected for 3-days when compared to uninfected WT mice (**Figure 3**). There was no significant increase in alveolitis, peribronchiolar inflammation and inflammatory cells in the lungs of infected NOX4 TG mice after three days compared to the infected NOX4 TG mice (**Figure 3**). However, at 7-days post infection there was a significant increase in alveolitis, peribronchiolar inflammation and inflammatory cells in the lungs of both WT mice and NOX4 TG mice that were similar in magnitude (**Supplementary Figure 1**).

### Airway Inflammation and Cell Differentials

**Figure 4:**
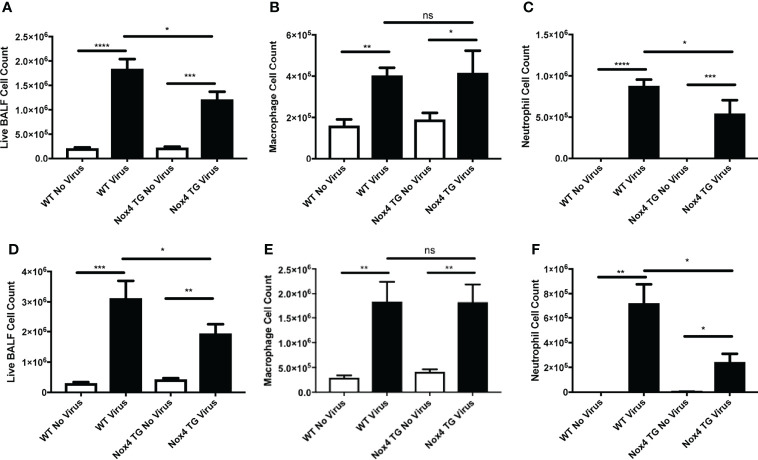
Endothelial NOX4 overexpressing mice infected with IAV had a decrease in airway inflammation compared to infected wild type mice. WT and NOX4 TG mice were infected with HK X-31 (10^4^ PFU) and total live cells, macrophages and neutrophils counted in BALF 3 **(A–C)** and 7 **(D–F)** days post infection. Data shown as mean ± S.E.M and analysed using one-way ANOVA with a Tukey Kramer post hoc test. (n = 8-15). *P < 0.05; **P < 0.01; ***P < 0.001, ****P < 0.0001, ns represents not-significant.

To evaluate airway inflammation, the total number of live cells in the BALF were counted. IAV infection of WT mice resulted in a substantial increase in BALF cell counts at Day 3 post-infection (~1.8x10^6^ cells) compared to uninfected WT mice (~2.1x10^5^ cells; **Figure 4A**). This response was further enhanced at 7 days post-infection (~3.1x10^6^ cells) compared to uninfected WT mice (~3.0x10^5^ cells; **Figure 4D**). In contrast, whilst IAV infection increased BALF cell counts in the NOX4 TG mice compared to the NOX4 TG uninfected group, the magnitude was significantly smaller than that observed in the WT mice at both Day 3 post infection (**Figure 4A**) and Day 7 post infection (**Figure 4D**).

We further characterised the infiltrating immune cells based on their morphology. An examination of specific cell types in the BALF showed a significant increase in macrophage and neutrophil infiltration in WT mice infected with IAV at both 3 and 7 days post infection (**Figure 4**). There was no significant difference between NOX4 TG and WT mice in macrophage infiltration 3 and 7 days post infection (**Figures 4B, E**). However, the degree of neutrophil infiltration in infected NOX4 TG mice at 3- and 7-days post infection was significantly lower than in the WT mice (**Figures 4C, F**).

### NOX2-Derived ROS Production

**Figure 5:**
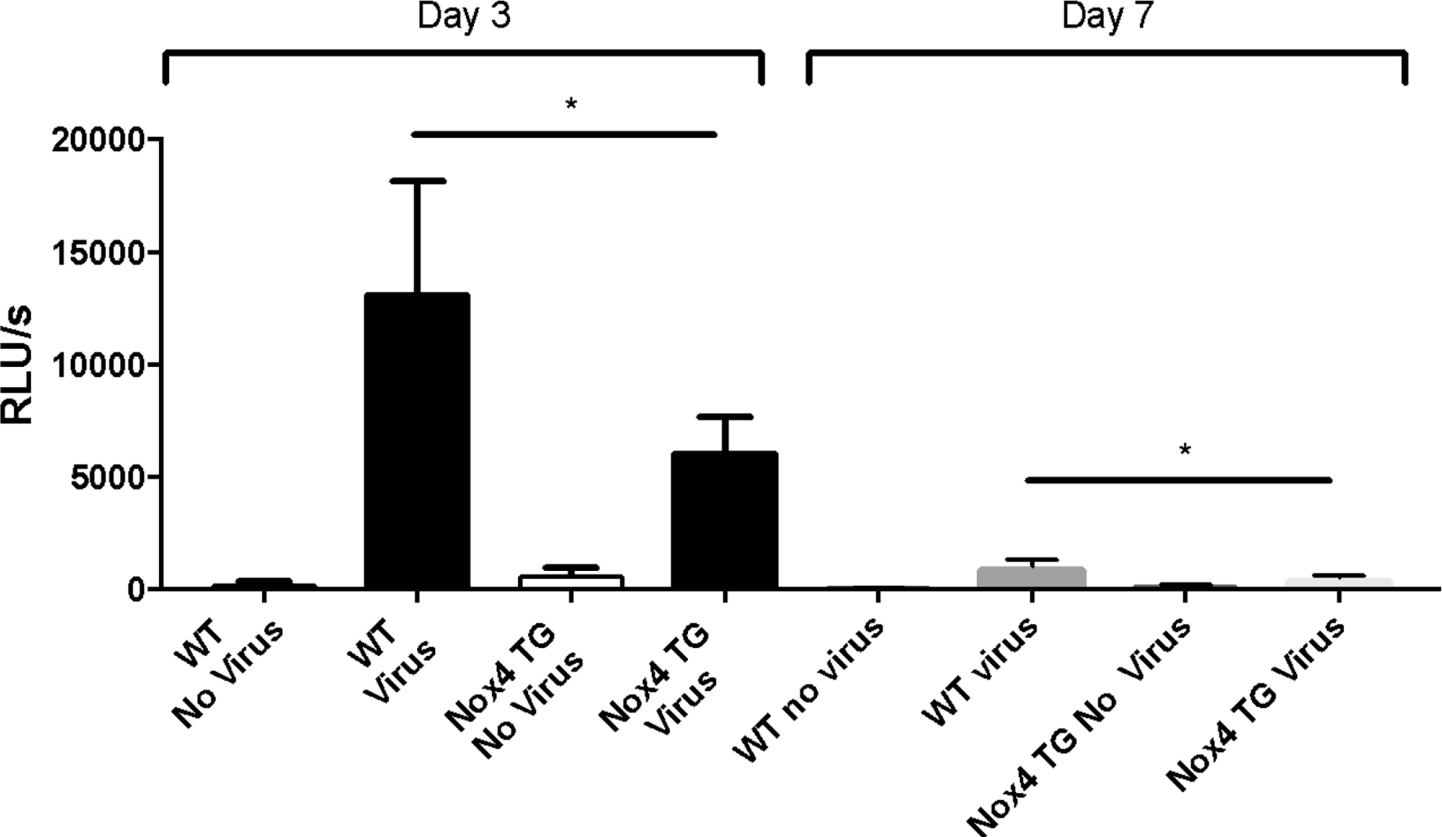
Endothelial NOX4 overexpression results in a decrease in IAV-induced BALF cell NOX2-derived ROS production. Fifty thousand BALF cells were extracted from HK x-31-infected (10^4^ PFU) WT and NOX4 TG mice at 3- and 7-days post infection and seeded in triplicated into a 96 well plate. Extracellular ROS production in these cells were measured with an L-012 enhanced chemiluminescence assay. Data are expressed as relative light units (RLU), measured as an average of triplicates over a 60-cycle period, subtracted by blank readings. Data shown as mean ± S.E.M and analysed using two-way ANOVA with a Tukey Kramer post hoc In review test. (n = 7-15) (*p < 0.05).

IAV infection of WT mice resulted in a significant increase in NOX2-derived ROS production by BALF inflammatory cells, as measured by L-012 chemiluminescence compared to uninfected mice at 3-days post infection (**Figure 5**). This ROS response also was observed at 7-days post infection, although the magnitude of this response was smaller than at Day 3 (**Figure 5**). Whilst there was also a significant increase in ROS production to IAV infection in NOX4 TG mice at 3- and 7-days post infection, the magnitude of this response was significantly lower than that observed in WT mice (**Figure 5**).

### Lung Chemotactic Cytokine Expression

**Figure 6:**
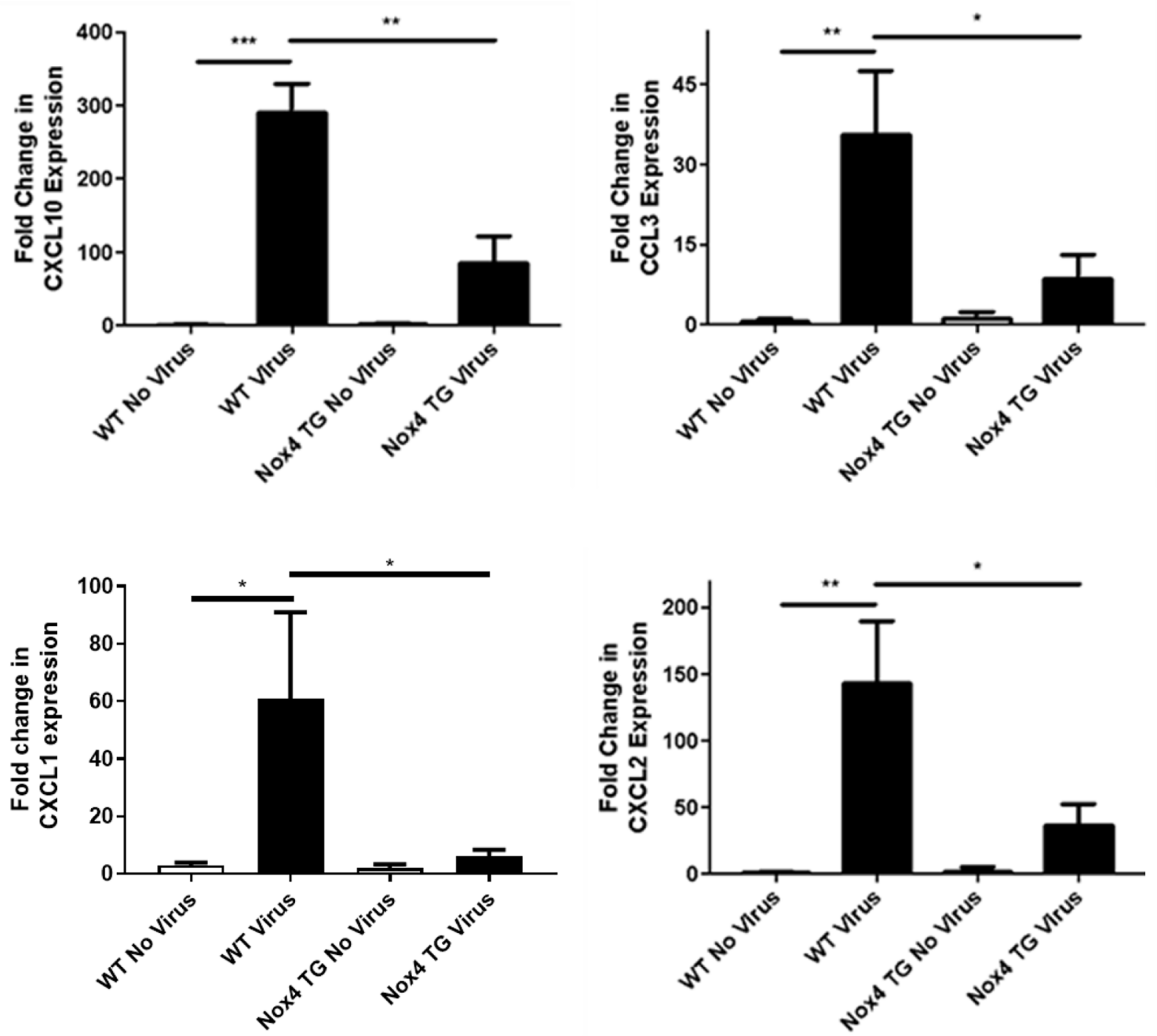
There was a significant decrease in CXCL10, CCL3, CXCL1 (KC) and CXCL2 expression in NOX4 overexpressing mice infected with influenza A virus 3-days post infection.WT and NOX4 TG mice were infected with HKX-31 (10^4^ PFU) for 3-days and lung mRNA expression of CXCL10, CCL3, CXCL1 (KC) and CXCL2 was measured. Responses are relative to 18S and is relative to the WT no virus controls. Data shown as mean ± S.E.M and analysed using one-way ANOVA with a Tukey Kramer post hoc test (n = 8-15) (p < 0.05). * P < 0.05; ** P < 0.01; *** P < 0.001.

We have thus far shown significant decreases in immune cell infiltration into the airway in NOX4 TG mice. Chemotactic factors play an important part in the inflammatory response, and we therefore examined whether the overexpression of NOX4 regulates this response to control airway inflammation. There was a significant increase in mRNA expression of macrophage and neutrophil chemotactic factors CXCL10, CCL3, CXCL1 (KC) and CXCL2 in the lungs of IAV-infected WT mice 3-days post infection. The magnitude of this response to IAV in NOX4 TG mice was significantly less (**Figure 6**).

## Discussion

Epithelial and inflammatory cells are critically involved in viral pathogenesis, but there is evidence that endothelial cells are also important orchestrators of the inflammatory response to IAV infection (Teijaro et al., 2011). ROS are also important mediators of IAV pathology, however, the role of ROS production by the endothelium in response to IAV infection has not previously been established. NOX4 is the most abundantly expressed isoform of the NOX enzyme family in lung endothelial cells and the expression of this, ROS producing enzyme was specifically suppressed in response to influenza infection. In NOX4 transgenic mice the expression of this enzyme and production of H_2_O_2_ is elevated by 2-3 fold (Schroder et al., 2012). Influenza A virus infection of these NOX4 TG mice resulted in a significantly attenuated airway and lung inflammatory response, reduced neutrophil infiltration, lower neutrophil chemotactic factor expression and a reduction in viral burden when compared to WT mice. Interestingly, this protective effect of NOX4 overexpression in the lung endothelium appeared to occur mainly during the early phases of the infection. This demonstrates that IAV infection suppresses lung endothelial NOX4 expression to promote an inflammatory response, which enables enhanced viral replication and exacerbated lung pathogenesis.

In some respects, our findings contrast with Amatore et al. (2015) who found that mucoepidermoid pulmonary carcinoma cells infected with the highly pathogenic PR8 influenza strain *in vitro* had increased NOX4 gene and protein expression. While the anti-NOX4 antibody used by Amatore et al. (2015) has been invalidated and raises questions about these findings (Altenhofer et al., 2012) it is important to consider that the infected cells were tumorigenic, which could have influenced NOX4 expression. In contrast, we examined lung NOX4 expression in an *in vivo* model, over multiple days and with a different strain of virus demonstrating significant reduction in NOX4 expression following IAV infection. However, it should be noted that the specific effects of IAV infection on NOX4 expression in cells other than the epithelium is largely unknown and that basal NOX4 expression in epithelial cells is low, particularly when compared to NOX2 and NOX1 (Kolarova et al., 2010). Given the low basal expression of NOX4 in the epithelial cells, the role of NOX4 in the endothelium may be more clinically relevant and a counter balance for the effects of the other NOX enzymes. Consequently, the decrease in NOX4 expression in the lungs of infected mice might reflect the pathogenic effects of NOX2 oxidase and the potential reciprocal regulation of these two NOX isoforms in vascular endothelial cells that requires further investigation. For instance, the silencing of NOX2 in lung endothelial cells has been shown to increase NOX4 expression and vice versa (Pendyala et al., 2009). A similar mechanism could be responsible for the decrease in NOX4 and it is also important to consider that this expression may be variable over the course of the infection. Moreover, nitric oxide has been shown to cause down regulation of NOX2 in human endothelial cells (Duerrschmidt et al., 2006), while H_2_O_2_ drives eNOS production in endothelial cells (Thomas et al., 2002). Since these NOX4 TG mice are producing ~2-3 times more hydrogen peroxide than WT mice, this could indicate an increase in NO production and, subsequently, NOX2 downregulation.

Weight loss was one of the indicators of morbidity assessed in this study and while all mice lost weight when infected with HK x-31, the NOX4 TG mice lost less weight than infected WT mice at the earlier time point after infection. This suggests that endothelial NOX4 has protective effects in the earlier stages of IAV infection. Additionally, there were similar findings with the other markers of morbidity, including lung weight, which was used as an indicator of pulmonary oedema and inflammation. WT mice infected with IAV had significantly increased lung weight, however, IAV did not significantly increase the lung weight of infected NOX4 TG mice. These findings suggest that NOX4 reduces pulmonary oedema and given its location at the endothelium this is a critical site to regulate this pathological process.

This study also examined the oxidative burst in lung inflammatory cells, which is solely due to NOX2 oxidase (Vlahos et al., 2011; Selemidis et al., 2013; Yatmaz et al., 2013; To et al., 2017). There was an increase in this extracellular NOX2 oxidase ROS production from the BALF cells of infected WT mice, which was significantly less in the NOX4 TG mice. NOX4 has been shown to drive the production of the antioxidant heme oxygenase 1 (HO1) (Schroder et al., 2012), which has protective effects in a mouse model of lung injury (Fredenburgh et al., 2007). This could account for the decrease in ROS production and the reduced lung weight in virus infected NOX4 TG mice. Alternatively, the reduction in ROS production in the NOX4 TG mice might be due to a reduction in the proportion of neutrophils in the BALF of those mice. Neutrophils are likely to significantly affect the overall ROS production of BALF inflammatory cells as they express high levels of NOX2 and drive an oxidative burst *via* this enzyme. For instance, the BALF from uninfected mice primarily consists of alveolar macrophages, whereas the BALF infected mice have a higher percentage of neutrophils, which could explain the differences in ROS production, owing to their greater capacity for NOX2 dependent ROS production. Compared to the infected WT mice, BALF from the infected NOX4 TG mice had a decreased percentage of neutrophils and this decrease in neutrophil infiltration could explain the decreased overall ROS levels seen in the BALF. Interestingly, ROS production at Day 7 was significantly lower than that observed at Day 3 for both WT and NOX4 TG mice, even though there were significant numbers of neutrophils still in the BALF. The marked reduction in ROS might be due to the relative lack of influenza virus detected at Day 7, and thus a reduction in stimulation of ROS generation. At Day 7 there is a significant adaptive immune response that ultimately clears the virus.

A hallmark feature of severe influenza infection is an excessive and detrimental inflammatory response (Dominguez-Cherit et al., 2009; Mauad et al., 2010). This study found an increase in airway inflammatory cell infiltration, alveolitis, peribronchiolar inflammation and overall lung inflammation in WT influenza-infected mice at both 3- and 7-days post infection. However, there was less airway inflammatory cell infiltration, alveolitis and peribronchiolar inflammation and overall inflammation in the lungs of infected NOX4 TG mice, 3-days post infection. This decrease in inflammation corresponded with reduced weight loss, lung weight and viral titres in infected NOX4 TG mice at day three. This demonstrated that enhanced ROS production from NOX4 oxidase has the capacity to attenuate the viral load and decrease the downstream pathogenesis caused by IAV infection at early phases of infection, however, additional mechanisms driving inflammation beyond Day 3 appear not to be influenced by endothelial NOX4.

Influenza infected NOX4 TG mice had decreased expression of CXCL10, CCL3, CXCL1 and CXCL2 three days post infection. The activation of CXCR3 by CXCL10 triggers influenza-induced neutrophil infiltration, and the deletion of CXCL10 and CXCR3 results in increased survival (Ichikawa et al., 2013). The decrease in CXCL10 expression in the influenza-infected NOX4 TG mice compared to infected WT mice is consistent with both the decrease in neutrophil infiltration and the decrease in lung weight seen in infected NOX4 TG mice. CXCL10 also contributes to the oxidative burst from neutrophils extracted from an acid-triggered acute lung injury (Ichikawa et al., 2013). This is consistent with the L-012 data in this study, which showed a decreased oxidative burst in infected NOX4 TG mice compared to the infected WT mice. CCL3 and CXCL2 have also been shown to be involved in the recruitment of neutrophils in models of bacterial infection (Zeng et al., 2003; De Filippo et al., 2013), and are both upregulated in influenza infection (Wareing et al., 2004). The downregulation of CCL3 in infected NOX4 TG explains the decrease in neutrophil infiltration. CCL3 and CXCL10 are known to trigger the infiltration of macrophages in influenza infection (Zeng et al., 2003; Ichikawa et al., 2013) yet, despite the decrease in these chemokines, there was no change in the number of macrophages in the infected NOX4 TG. Either these are redundant pathways, or other compensatory mechanisms are involved in monocyte/macrophage infiltration. It is interesting that the early effects on inflammation were more pronounced than at later time points suggesting that the viral infection eventually overrides the early effects of NOX4 ROS production presumably as the cytokine response gains momentum. Of importance, these cytokines aforementioned are involved in regulating the recruitment of both macrophages and neutrophils. Specifically, a reduction in virus-induced neutrophilic inflammation is likely explained by a reduction in the expression of CXCL1 (KC), a chemoattractant that mediates the recruitment and trafficking of neutrophils to the site of infection (De Filippo et al., 2013; Sawant et al., 2015). In corroboration with our observations, CXCR1/2 antagonism alleviated neutrophilia and pulmonary damage in mice challenged with streptococcus pneumoniae and influenza virus, highlighting the selective nature of the chemoattractant CXCL1 in modulating neutrophilic inflammation associated immunopathology (Tavares et al., 2017).

The seemingly opposing roles of NOX2 and NOX4 in IAV pathogenesis aided by the observational nature of this study, enables a series of new hypotheses and questions that warrant further experimentation to be proposed. For example, is there any relationship in the kinetics of NOX2 and NOX4 and their resulting effects on the immune response to IAV? What are the ratios of NOX2 and NOX4 expression at various stages of the course of IAV infection and is NOX4 activated following the inflammation by NOX2 to mediate control during an IAV infection? Also, given the different subcellular compartmentalisation of NOX2 and NOX4, i.e. endosomal Vs endoplasmic reticulum/mitochondria, is there any cross regulation such that one might provide a dominant response over the other in a temporal fashion to instil pro-inflammatory dominant vs anti-inflammatory response from the endothelium? It must be kept in mind though, that NOX2 expression is much higher than NOX4 in inflammatory cells such as macrophages and neutrophils, and is likely to dominate the inflammatory response in these cells. Whilst NOX4 expression could be under the influence of NOX2, there might be additional factors that regulate NOX4 expression including factors that influence p22phox. Downregulation of p22phox can lead to destabilisation of the NOX4-p22phox heterodimer and thereby suppression of NOX4 activity. A better understanding of the dynamics of NOX2 and NOX4 activity during IAV infection is important for future therapeutic development strategies that aim to target oxidative stress pathways. Indeed, these fundamental differences in roles of NOX4 and NOX2 would strongly suggest that broad based anti-oxidants are unlikely to be of benefit but rather a more targeted spatial-temporal approach is necessary to address IAV mediated ROS production.

In summary, NOX4 has been shown to be protective against inflammatory and ischemic stress. This study has shown for the first time that endothelial NOX4 also ameliorates some symptoms of IAV infection, including oxidative stress and neutrophil infiltration, lung damage, and cytokine expression. This study further highlights the role of the endothelium in IAV pathology and the opposing roles of NOX isoforms in this context. This exemplifies the current paradigm that ROS production is not always detrimental, and that the specific site of ROS production can have vastly different effects on pathogenesis. It appears that while endosomal ROS production drives pathogenesis, that of NOX4 derived ROS production has the capacity to attenuate it. Future studies are needed to decipher the subcellular roles of endothelial NOX4, in particular, its expression in mitochondria places it at a site that might regulate mitochondrial ROS levels and thereby inflammation. Consequently, the specific pharmacological induced increase of endothelial NOX4 activity could be a means of reducing IAV-induced inflammation and oxidative stress and improve patient outcomes by suppressing the viral replication and downstream pathogenesis.

## Data Availability Statement

The raw data supporting the conclusions of this article will be made available by the authors, without undue reservation.

## Ethics Statement

The animal study was reviewed and approved by Monash University Animal Ethics Committee.

## Author Contributions

KH, ET, RL, FL, JE, and SL performed experiments. KH, ET and SS wrote the manuscript KH, ET, RL, FL, SL, JE, AS, JO’L, DB, RV, and SS provided intellectual input and edited the manuscript. SS, RV, DB, and JO’L were involved in conceiving and funding this research project. SS supervised and managed the overall study. All authors contributed to the article and approved the submitted version.

## Funding

This work was supported by the Australian Research Council (ARC) Future Fellowship Scheme for SS (I.D. FT120100876) and The National Health and Medical Research Council of Australia (NHMRC Project I.D. 1122506, 1128276).

## Conflict of Interest

The authors declare that the research was conducted in the absence of any commercial or financial relationships that could be construed as a potential conflict of interest.

## Publisher’s Note

All claims expressed in this article are solely those of the authors and do not necessarily represent those of their affiliated organizations, or those of the publisher, the editors and the reviewers. Any product that may be evaluated in this article, or claim that may be made by its manufacturer, is not guaranteed or endorsed by the publisher.
