# Supplementary material for: Endothelial NOX4 Oxidase Negatively Regulates Inflammation and Improves Morbidity During Influenza A Virus Lung Infection in Mice
Source: Front Cell Infect Microbiol. 2022 May 4;12:883448. doi: 10.3389/fcimb.2022.883448 (PMC9115386; doi:10.3389/fcimb.2022.883448)
Supplement: Supplementary file 1 [file Image_1.pdf]

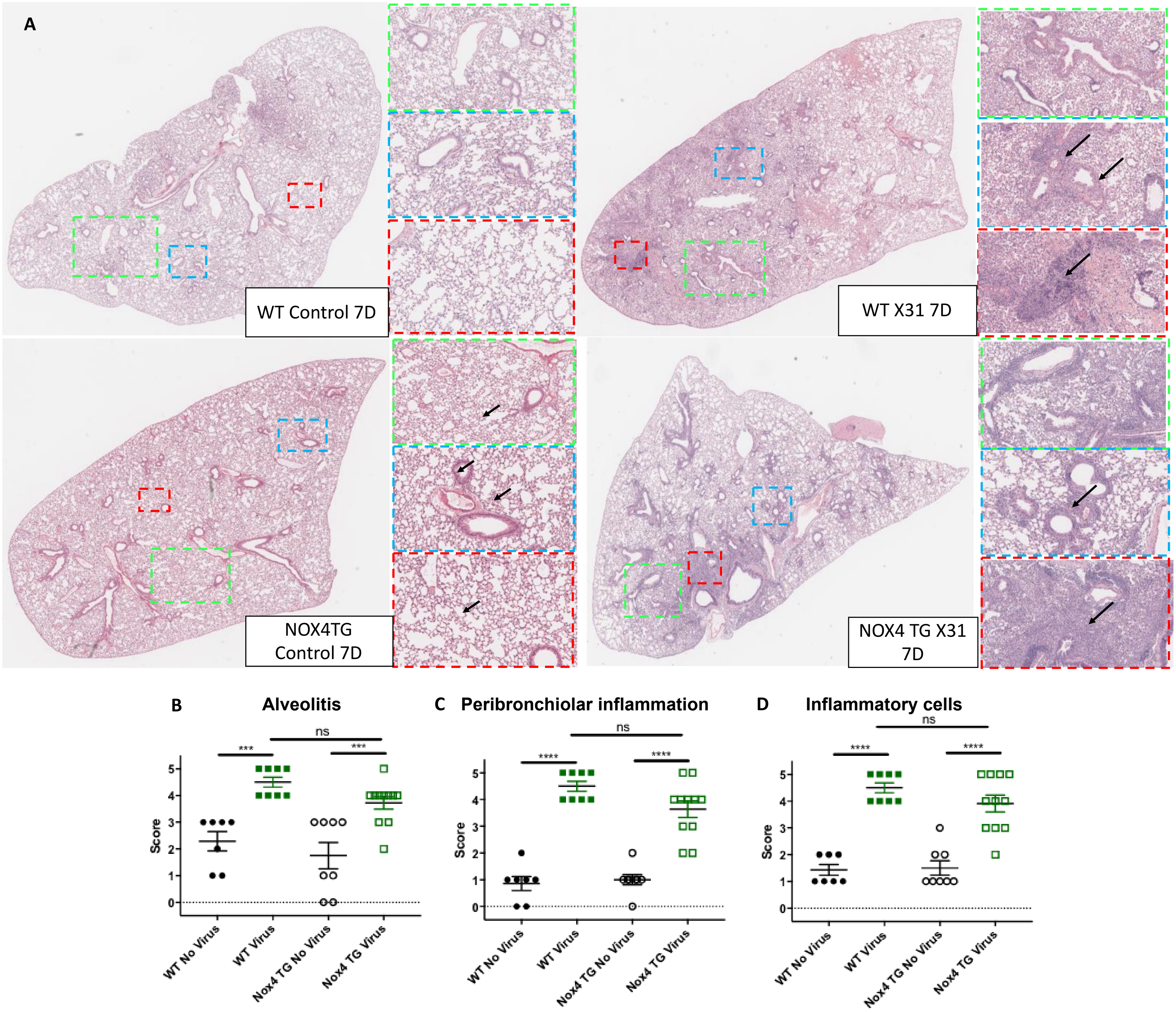


**Supplementary Figure 1. There was no significant difference in alveolitis, peribronchiolar inflammation and inflammatory cells in the lungs of influenza-infected NOX4 TG mice compared to infected WT mice seven days post infection.**

**A.** Hematoxylin and eosin (H&E) stained paraffin sections of lungs of WT and NOX4 TG mice 7-days post infection with HK x-31 ($10^4$ PFU) or treatment with PBS. **B**. Alveolitis, **C**. peribronchiolar inflammation and **D.** inflammatory cell infiltration. Representative images displaying the inflammation in lung that were sectioned longitudinally following H&E staining. The arrows within the blue areas indicate the peri-bronchial inflammation and in the red areas they indicate the alveolitis. Each sample was scored blindly from 0-5 for each individual mouse (higher numbers indicate increased disease severity) from two independent assessors. The representative images were obtained at a x40 magnification using the Aperio Slide Scanning Unit. The images were then viewed with the Aperio image software and different areas of the lung section were subsequently digitally zoomed as shown in the figure. The three different digital magnifications are x1 (green), x3 (blue) and x6(red). Data shown

as mean ± s.e.m and analysed using a non-parametric one-way ANOVA with a Kruskal-Wallis *post hoc* test (n=7-12) ($p<0.05$). *** $P<0.001$, **** $P<0.0001$, ns represents not significant.
